# The effect of biochanin A on the antibiotic susceptibilities of *Escherichia coli* grown under different respiration and metabolic conditions on agar media

**DOI:** 10.1186/s12866-026-04794-2

**Published:** 2026-03-17

**Authors:** Jourdan E. Lakes, Jessica L. Ferrell, Michael D. Flythe

**Affiliations:** 1 USDA Agricultural Research Service Forage-Animal Production Research Unit, 1100 S. Limestone St., Lexington, KY 40516 USA; 2 Department of Animal and Food Sciences, College of Agriculture, Food and Environment, University of Kentucky, Lexington, KY USA

**Keywords:** Escherichia coli, Biochanin a, Multi-drug resistance, Antibiotic resensitization

## Abstract

**Background:**

Antimicrobials have been used for a number of purposes in beef cattle production. However, increased prevalence of pathogens, including zoonotic bacterial species, such as *Escherichia coli*, is a concern. The flavonoid, biochanin A (BCA), was identified as a natural feed component to potentially limit the spread of antibiotic-resistant microorganisms by potentiating the activity of other antimicrobials. The current study investigated the synergistic effects of 120 ppm BCA with five classes of antibiotics on two strains of *E. coli*: 25922 and 51659 (O157:H7) on two agar culture media by using commercially available ETEST^®^ strips and the FDA/CLSI breakpoint interpretation criteria.

**Results:**

The effect of 120 ppm BCA on minimum inhibitory concentration (MIC) across the antibiotic classes was relatively consistent where it had largely no effect, an occasionally negative effect, or a neutral effect. BCA addition yielded negligible changes to ciprofloxacin (susceptible) for either strain under the redox and metabolic conditions tested. The culture conditions seemed more influential over MIC than BCA. The absence of oxygen generally conferred clinical resistance or a higher MIC, and at times media composition altered the MICs as well. For example, neither macrolide inhibited either strain (> 256 µg mL^− 1^) under anaerobic conditions but generally had measurable MICs aerobically. Additionally, the MICs for streptomycin were consistently higher across each strain when cultured on tryptic soy agar rather than Mueller Hinton broth.

**Conclusions:**

Despite previous studies in which biochanin A decreased MICs in other bacterial species, neither strain of *E. coli* was consistently impacted by the presence of 120 ppm BCA in the culture medium. The data indicate that BCA is likely not a reliable resensitizing/synergistic candidate for resistant *E. coli*. Regardless, the data demonstrates the impact culture conditions have on MIC across antibiotic class. These differences in susceptibility may be necessary to consider when treating *E. coli* infections under different conditions.

## Background

As the world’s population continues to increase and food demand rises, the necessity for food production efficiency becomes rather pertinent, and one such place for increased efficiency is in beef production. Cattle feedlot operations are an integral part of this system by reducing the necessary time and cost to obtain finished cattle via alterations to the diet [1]. However, the improper transition of feeder cattle from a high forage diet to a traditional feedlot diet (high concentrate/grain) is associated with harmful and damaging environmental changes to the rumen, such as significant decreases in ruminal pH which contributes to the occurrence of parakeratosis, rumenitis, and ultimately pathogen infiltration in the rumen [2]. Due to the increased incidence of pathogen occurrence in feedlot cattle, antimicrobials are often supplemented into the feed to prevent disease, such is the case with Fusobacteria-related liver abscesses and Tylan^®^ (a macrolide) antibiotic treatment [3, 4]. Any use of antimicrobials increases the risk of antimicrobial resistance (AMR) adaptations in pathogens, many of which are zoonotic and pose risk to human populations [5].

One such zoonotic pathogen transmissible between livestock and humans is enterohemorrhagic *Escherichia coli* [6]. Importantly, this organism is known to carry AMR genes for a wide range of antimicrobials. Isolates from animals in particular demonstrate resistance to the tetracyclines, which are commonly used in both livestock and human populations [7, 8]. In fact, previous works determined that subtherapeutic antimicrobial application increased the incidence of tetracycline-resistant *E. coli* in feedlot cattle, and that *E. coli* O157:H7 is, indeed, capable of surviving in strong acidic environments, such as the gastrointestinal systems of cattle fed high grain diets [9, 10]. These observations underscore the importance of either identifying novel sources of antimicrobials or compounds capable of resensitizing multi-drug-resistant zoonotic pathogens, such as *E. coli*, to existing antimicrobials. Resensitizing agents act by inhibiting bacterial methods of resistance, e.g., activity or synthesis of efflux pumps, enzymes, biofilm formation, and quorum-sensing [11]. Thus, resensitizing compounds often increase the effectiveness of pre-existing antimicrobial agents, lessening the burden on novel antimicrobial discovery.

Previous observations by our group demonstrated that the number of ruminal tetracycline-resistant organisms could be substantially reduced in vivo by addition of biochanin A (BCA), an isoflavone of red clover *Trifolium pratense*, in a loose mineral fed to steers on a high grain diet [12]. Similarly, other groups demonstrated successful resensitization to tetracycline in vitro by *Staphylococcus epidermidis* strains using essential oils from *Salvia* spp [13]. Given the existing evidentiary support for *E. coli* prevalence in feedlot cattle often treated with antimicrobials, this work sought to investigate BCA as a possible resensitizing or synergistic agent of *E. coli* to several tetracyclines and other antibiotic classes.

## Methods

### Bacterial strains

The test strain *E. coli* O157:H7 (ATCC 51659) and reference strain *E. coli* (ATCC 25922) were purchased from the American Type Culture Collection (ATCC; Manassas, VA, USA). Strain 51659 was maintained in tryptic soy broth (TSB) at 37 °C per the ATCC guidelines. Strain 25922 was reconstituted in TSB and incubated at 37 °C overnight. Following incubation, a glycerol stock of the stationary phase 25922 culture was prepared (2 mL culture in 1.5 mL glycerol stock vial) and stored at −20 °C until use. Prior to experimentation, 25922 was removed from freezer storage, inoculated into fresh TSB and optical density (600 nm; OD_600_) adjusted prior to plating on tryptic soy agar (described below). Routine passage of 25922 in broth is discouraged by ATCC as it has been associated with minimum inhibitory concentration (MIC) variability during susceptibility testing [14].

### Culture media

TSB powder was purchased from BD Difco™ (Franklin Lakes, NJ, USA) for the culture of *E. coli* per ATCC recommendations. The composition of TSB contains (per 1 L): 17 g pancreatic digest of casein, 3 g papaic digest of soybean, 2.5 g dextrose, 5 g NaCl, and 2.5 g K_2_PO_4_ at pH 7.3 ± 0.2. Briefly, 30 g of TSB powder was dissolved in 1 L of DI water and autoclaved at 121 °C for 15 min and either dispensed aerobically on the benchtop or anaerobically in an anaerobic chamber (gas mix of 95% CO_2_/5% H_2_) in 18 × 150 mm borosilicate glass tubes with chlorobutyl 20 mm stoppers and aluminum crimp caps. Dispensed and tubed broth was then autoclaved for sterility at the same parameters as above.

Tryptic soy agar (TSA; BD Difco™ Franklin Lakes, NJ, USA) contains all elements of TSB with the following exceptions: no K_2_PO_4_, and instead 15 g agar. TSA was prepared per 1 L according to manufacturer instructions: 40 g of TSA powder was dissolved in 1 L of DI water, boiled for 1 min, then autoclaved at 121 °C for 15 min. Autoclaved TSA was allowed to cool (prior to agar beginning to set) and poured either aerobically in a sterilized biosafety cabinet or anaerobically in the anaerobic chamber (as above) into sterile 15 × 100 mm petri dishes.

Mueller Hinton agar (MHA) was prepared by suspending 22 g of Mueller Hinton II Cation-Adjusted Broth (BD Difco™ BBL™, Franklin Lakes, NJ, USA) and 17 g of agar per 1 L of DI water, boiling for 1 min, then autoclaving at 121 °C for 15 min. The autoclave agar medium was cooled to touch-temperature and poured in the same manner as TSA into 15 × 100 mm petri plates. MHA contained per 1 L, the following: 3 g beef extract, 17.5 g acid hydrolysate of casein, 1.5 g starch, and 17 g agar (pH 7.3 ± 0.1).

### Minimum inhibitory concentration testing

The MICs of *E. coli* strains 51659 and 25922 were determined with or without 120 ppm BCA for tetracycline, minocycline, doxycycline, azithromycin, erythromycin, ciprofloxacin, trimethoprim/sulfamethoxazole, trimethoprim, and streptomycin using the ETEST^®^ (bioMérieux SA, Marcy-l’Etoile, France). A 6,000 ppm (6 mg ml^−1^) stock solution of biochanin A (Cayman Chemical, Ann Arbor, MI, USA) was prepared in 200 proof filter-sterilized ethanol (stored at −20 °C) and used at a working concentration of 120 ppm. This concentration of BCA was chosen for the study, of those tested (30, 60, and 120 ppm), as this concentration is comparable with those of previous works [15, 16] and did not precipitate upon addition to the still liquid agar, which was observed at higher attempted concentrations. It should be noted that neither 30 nor 60 ppm BCA concentrations were antimicrobial, whereas 120 ppm showed slight antimicrobial activity, which we concluded was ideal for synergy testing (data not shown). The optical density (measured at 600 nm; OD_600_) of stationary phase (~ 24 h) 51659 and reconstituted 25922 cultures were adjusted with sterile 0.85% NaCl solution to approximately 0.5 OD_600_ and 200 µl of the adjusted culture was spread onto the surface of one of the following plate preparations: MHA, MHA + 120 ppm BCA, TSA, and TSA + 120 ppm BCA. The inocula were titered to verify that the density of viable cells within the suspensions were within the acceptable range of 1 to 5 × 10^8^ CFU ml^− 1^ (approximate McFarland equivalent of 0.5). Once the inoculated agar surface was dry, two test strips were laid onto each plate following the template for 2 strips per 90 mm plate as described by the manufacturer. Plates were inverted and incubated at 37 °C for 20 h either aerobically or anaerobically (95% CO_2_/5% H_2_). Following incubation, the point at which the zone of growth inhibition intersected (as specified by examples on the package insert for each antibiotic ETEST^®^) the strip was read as the MIC of each antibiotic and interpreted according to FDA/CLSI breakpoints (CLSI M100: Performance Standards for Antimicrobial Susceptibility Testing). All experiments were performed at least three times using biological replicates.

While MHA is the recommended medium by CLSI for antimicrobial susceptibility testing, TSA was also included in this study to capture potential media-dependent changes in MIC that could have been attributed to slight differences in the nutritive factors of each medium, e.g., peptides, amino acids, vitamins, carbohydrates, and minerals. It should also be noted that while MHA is cation adjusted (calcium and magnesium, specifically), the calcium and magnesium content is uncontrolled in TSA [17], which has been shown to increase the MICs of tetracycline for *E. coli* 25922 cultured aerobically [18].

## Results

The control strain 25922 was susceptible to the tetracyclines regardless of exposure to oxygen (aerobic versus anaerobic), media type (MHA versus TSA), or BCA addition according to established CLSI breakpoints (Tables 1, 2 and 3). Whereas the toxigenic strain 51659 was not inhibited by any concentration of tetracycline (0.016–256 µg mL^− 1^) under the testing conditions (Table 1) and was variably sensitive to minocycline and doxycycline (Tables 2 and 3, respectively). For example, 51659 was largely resistant to minocycline in MHA apart from the anaerobic control (sans BCA) where it was intermediately sensitive but lost this sensitivity when BCA was added to the medium (Table 2). However, this observation reversed in TSA, where the anaerobic control was resistant to minocycline and became intermediately sensitive with the addition of BCA. Similarly to tetracycline, 51659 was not inhibited by doxycycline under the majority of the testing conditions (Table 3). However, when 51659 was cultured aerobically in TSA, there were quantifiable MICs, but these values were still considered clinically resistant.

**Table 1:** *E. coli* tetracycline minimum inhibitory concentrations in agar with or without 120 ppm biochanin A

		Mueller Hinton Agar				Tryptic Soy Agar			
*E. coli* strain	Treatment	MIC^a^ Aerobic	CLSI Result^b^	MIC Anaerobic	CLSI Result	MIC Aerobic	CLSI Result	MIC Anaerobic	CLSI Result
ATCC 25922	Control	2 (2)	S	0.5 (0.38–0.5)	S	4 (4)	S	2 (1.5–2.5)	S
	120 ppm Biochanin A	1.5 (1.5)	S	0.25 (0.25)	S	2 (2)	S	1.5 (1–1.5.5)	S
O157:H7 ATCC 51659	Control	> 256	R	> 256	R	> 256	R	> 256	R
	120 ppm Biochanin A	> 256	R	> 256	R	> 256	R	> 256	R

* S* susceptible (≤ 4), *I * intermediate (8), *R* resistant (≥ 16)

^a^ Median MIC of antimicrobial range tested (0.016–256 µg mL^− 1^)

^b^ CLSI breakpoint interpretation

**Table 2:** *E. coli* minocycline minimum inhibitory concentrations in agar with or without 120 ppm biochanin A

		Mueller Hinton Agar				Tryptic Soy Agar			
*E. coli* strain	Treatment	MIC^a^ Aerobic	CLSI Result^b^	MIC Anaerobic	CLSI Result	MIC Aerobic	CLSI Result	MIC Anaerobic	CLSI Result
ATCC 25922	Control	1 (1)	S	0.25 (0.25)	S	2 (2)	S	0.5 (0.5–1.5)	S
	120 ppm Biochanin A	2 (2)	S	0.25 (0.25)	S	2 (1.5–2.5)	S	1 (1)	S
O157:H7 ATCC 51659	Control	> 256	R	6 (6)	I	32 (32)	R	> 256	R
	120 ppm Biochanin A	> 256	R	> 256	R	64 (32–64)	R	6 (6)	I

*S * susceptible (≤ 4), *I * intermediate (8), *R* resistant (≥ 16)

^a^ Median MIC of antimicrobial range tested (0.016–256 µg mL^− 1^)

^b^ CLSI breakpoint interpretation

**Table 3:** *E. coli* doxycycline minimum inhibitory concentrations in agar with or without 120 ppm biochanin A

		Mueller Hinton Agar				Tryptic Soy Agar			
*E. coli* strain	Treatment	MIC^a^ Aerobic	CLSI Result^b^	MIC Anaerobic	CLSI Result	MIC Aerobic	CLSI Result	MIC Anaerobic	CLSI Result
ATCC 25922	Control	2 (1.5–2.5)	S	0.75 (0.75)	S	3 (3–4)	S	2 (2)	S
	120 ppm Biochanin A	1.5 (1–2)	S	0.75 (0.75)	S	2 (2–3)	S	2 (1–2)	S
O157:H7 ATCC 51659	Control	> 256	R	> 256	R	48 (48–64)	R	> 256	R
	120 ppm Biochanin A	> 256	R	> 256	R	96 (96)	R	> 256	R

*S * susceptible (≤ 4), * I * intermediate (8), *R* resistant (≥ 16)

^a^ Median MIC of antimicrobial range tested (0.016–256 µg mL^− 1^)

^b^ CLSI breakpoint interpretation

While the breakpoint interpretations have not been established by CLSI, the MICs of 25922 and 51659 to two macrolides (azithromycin and erythromycin) were also evaluated in the presence or absence of 120 ppm BCA. When *E. coli* was grown on either MHA or TSA anaerobically, there was no apparent susceptibility at the concentrations tested (0.016–256 µg mL^− 1^) to either macrolide regardless of BCA addition (Tables 4 and 5). When grown aerobically on MHA with or without BCA in the presence of azithromycin, both 25922 and 51659 had MICs between 1 and 2 µg mL^− 1^, whereas the MICs on aerobic TSA increased (4 and 12 µg mL^− 1^; Table 4). Interestingly, the addition of BCA in aerobic TSA media increased the MIC of toxigenic *E. coli* 51659 exposed to both azithromycin (4 versus 12 µg mL^− 1^) and erythromycin (64 versus 128 µg mL^− 1^). Conversely, the addition of BCA in aerobic MHA media decreased the MIC of erythromycin from no observable inhibition to 16 µg mL^− 1^ (Table 5). Where 51659 exposed aerobically to erythromycin saw a reversal in the effect of BCA addition in the media between MHA and TSA, 25922 was more consistent in that BCA increased the MIC from 48 to 64 µg mL^− 1^ in both MHA and TSA media.

**Table 4:** *E. coli* azithromycin minimum inhibitory concentrations in agar with or without 120 ppm biochanin A

		Mueller Hinton Agar				Tryptic Soy Agar			
*E. coli* strain	Treatment	MIC^a^ Aerobic	CLSI Result^b^	MIC Anaerobic	CLSI Result	MIC Aerobic	CLSI Result	MIC Anaerobic	CLSI Result
ATCC 25922	Control	1 (1–1.5.5)	NE	> 256	NE	8 (8)	NE	> 256	NE
	120 ppm Biochanin A	1.5 (1.5)	NE	> 256	NE	12 (8–12)	NE	> 256	NE
O157:H7 ATCC 51659	Control	2 (1.5–2.5)	NE	> 256	NE	4 (4)	NE	> 256	NE
	120 ppm Biochanin A	1.5 (1–1.5.5)	NE	> 256	NE	12 (8–12)	NE	> 256	NE

^a^ Median MIC of antimicrobial range tested (0.016–256 µg mL^− 1^)

^b^ CLSI breakpoint interpretation not established (NE) for *E. coli*

**Table 5:** *E. coli* erythromycin minimum inhibitory concentrations in agar with or without 120 ppm biochanin A

		Mueller Hinton Agar				Tryptic Soy Agar			
*E. coli* strain	Treatment	MIC^a^ Aerobic	CLSI Result^b^	MIC Anaerobic	CLSI Result	MIC Aerobic	CLSI Result	MIC Anaerobic	CLSI Result
ATCC 25922	Control	48 (48)	NE	> 256	NE	48 (48)	NE	> 256	NE
	120 ppm Biochanin A	64 (64)	NE	> 256	NE	64 (64)	NE	> 256	NE
O157:H7 ATCC 51659	Control	> 256	NE	> 256	NE	64 (64)	NE	> 256	NE
	120 ppm Biochanin A	16 (16)	NE	> 256	NE	128 (128)	NE	> 256	NE

^a^ Median MIC of antimicrobial range tested (0.016–256 µg mL^− 1^)

^b^ CLSI breakpoint interpretation not established (NE) for *E. coli*

*E. coli* 25922 and 51659 were susceptible under all testing conditions to ciprofloxacin, a second-generation fluoroquinolone, according to the breakpoints established by CLSI (Table 6). There was an increase in MIC in TSA media when BCA was added for both aerobic and anaerobic preparations of 51659 but only in the aerobic preparation for 25922. Additionally, MIC was differentially impacted by BCA in aerobic (increased) and anaerobic (decreased) preparations of 51659 in MHA. However, the changes in MIC for any of the testing conditions did not change the CLSI interpretation of the results, namely both 25922 and 51659 were susceptible to ciprofloxacin.

**Table 6:** *E. coli* ciprofloxacin minimum inhibitory concentrations in agar with or without 120 ppm biochanin A

		Mueller Hinton Agar				Tryptic Soy Agar			
*E. coli* strain	Treatment	MIC^a^ Aerobic	CLSI Result^b^	MIC Anaerobic	CLSI Result	MIC Aerobic	CLSI Result	MIC Anaerobic	CLSI Result
ATCC 25922	Control	0.008 (0.008)	S	0.032 (0.032)	S	0.008 (0.008)	S	0.19 (0.19)	S
	120 ppm Biochanin A	0.012 (0.008–0.012)	S	0.032 (0.032)	S	0.016 (0.016)	S	0.19 (0.19)	S
O157:H7 ATCC 51659	Control	0.023 (0.016–0.023)	S	0.094 (0.094)	S	0.012 (0.012)	S	0.094 (0.094)	S
	120 ppm Biochanin A	0.032 (0.032)	S	0.064 (0.064)	S	0.023 (0.023)	S	0.19 (0.19)	S

*S* susceptible (≤ 0.25), * I * intermediate (0.5), *R * resistant (≥ 1)

^a^ Median MIC of antimicrobial range tested (0.002–32 µg mL^− 1^)

^b^ CLSI breakpoint interpretation

*E. coli* was very distinctly either susceptible or resistant to antifolates alone or in combination with a sulfonamide: trimethoprim/sulfamethoxazole or trimethoprim alone (Tables 7 and 8, respectively). *E. coli* 25922 was clinically susceptible to the antibiotic combination trimethoprim/sulfamethoxazole, when cultured aerobically in either MHA or TSA. However, while 25922 was originally susceptible anaerobically to the antibiotics in either medium, upon addition of BCA it was clinically resistant and displayed no inhibition at the concentrations tested (0.002–32 µg mL^− 1^; Table 7). Similarly, *E. coli* 51659 was susceptible to the antibiotic combination when cultured aerobically in both MHA and TSA, though the MIC increased upon addition of BCA. When 51659 was cultured anaerobically in MHA amended with BCA there was a distinct MIC increase, but it did not alter the CLSI breakpoint designation and remained clinically susceptible. Additionally, 51659 was resistant to trimethoprim/sulfamethoxazole when cultured anaerobically in TSA media with or without BCA and displayed no inhibition at the concentrations tested (Table 7). Without the addition of sulfamethoxazole, the control strain (25922) lost sensitivity to trimethoprim in TSA at the concentrations (0.002–32 µg mL^− 1^) and conditions tested (Table 8). However, in MHA regardless of the presence of BCA or oxygen, 25922 remained clinically susceptible to trimethoprim albeit at generally higher quantifiable MIC values than when the antibiotic was given with sulfamethoxazole. The MICs of 51659 in MHA or TSA exposed to trimethoprim with sulfamethoxazole were largely comparable to those without (trimethoprim only; Tables 7 and 8, respectively). This observation excludes 51659 grown anaerobically on MHA amended with BCA, where it was not inhibited by the concentrations of trimethoprim tested and thereby resistant according to established CLSI breakpoints (Table 8).

**Table 7:** *E. coli* trimethoprim/sulfamethoxazole minimum inhibitory concentrations in agar with or without 120 ppm biochanin A

		Mueller Hinton Agar				Tryptic Soy Agar			
*E. coli* strain	Treatment	MIC^a^ Aerobic	CLSI Result^b^	MIC Anaerobic	CLSI Result	MIC Aerobic	CLSI Result	MIC Anaerobic	CLSI Result
ATCC 25922	Control	0.094 (0.094–0.125)	S	0.094 (0.094)	S	0.25 (0.25–0.38)	S	0.25 (0.25)	S
	120 ppm Biochanin A	0.125 (0.125)	S	> 32	R	0.25 (0.25–0.38)	S	> 32	R
O157:H7 ATCC 51659	Control	0.25 (0.25)	S	0.19 (0.19)	S	0.19 (0.19)	S	> 32	R
	120 ppm Biochanin A	0.5 (0.5)	S	0.5 (0.38–0.5)	S	0.5 (0.5–1.5)	S	> 32	R

* S* susceptible (≤ 2), *I* intermediate (-), *R* resistant (≥ 4)

^a^ Median MIC of antimicrobial range tested (0.002–32 µg mL^− 1^)

^b^ CLSI breakpoint interpretation

**Table 8:** *E. coli* trimethoprim minimum inhibitory concentrations in agar with or without 120 ppm biochanin A

		Mueller Hinton Agar				Tryptic Soy Agar			
*E. coli* strain	Treatment	MIC^a^ Aerobic	CLSI Result^b^	MIC Anaerobic	CLSI Result	MIC Aerobic	CLSI Result	MIC Anaerobic	CLSI Result
ATCC 25922	Control	1.5 (1.5–2.5)	S	0.25 (0.25)	S	> 32	R	> 32	R
	120 ppm Biochanin A	3 (3)	S	0.75 (0.75)	S	> 32	R	> 32	R
O157:H7 ATCC 51659	Control	0.25 (0.25–0.38)	S	0.38 (0.38)	S	0.38, (0.38–0.5)	S	> 32	R
	120 ppm Biochanin A	0.5 (0.5–0.75)	S	> 32	R	1 (0.5–1.5)	S	> 32	R

* S* susceptible (≤ 8), * I * intermediate (-), * R* resistant (≥ 16)

^a^ Median MIC of antimicrobial range tested (0.002–32 µg mL^− 1^)

^b^ CLSI breakpoint interpretation

While a CLSI breakpoint for the aminoglycoside, streptomycin, on Enterobacterales using ETEST^®^ strips has not presently been established, the MICs under the testing conditions of this study are still reported. Strain 51659 was generally less susceptible (higher MIC values) to streptomycin than the control strain (Table 9). MIC values of both strains were greatly affected by anaerobic versus aerobic growth conditions, i.e., there was either no inhibition, or the MIC increased dramatically when cultured anaerobically in either MHA or TSA. For the control strain (25922) the addition of BCA to MHA media slightly decreased the MIC when grown both aerobically (1.5 versus 1 µg mL^− 1^) or anaerobically (32 versus 16 µg mL^− 1^). This observation reversed in TSA where BCA addition increased the MIC (3 versus 8 µg mL^− 1^ – aerobic, 64 versus no inhibition/>1024 µg mL^− 1^ – anaerobic). Alternatively, toxigenic *E. coli* strain 51659 appeared less susceptible to streptomycin when BCA was added to either MHA or TSA and incubated aerobically. However, when 51659 was cultured anaerobically, there was either a decrease in MIC (presumably increased susceptibility; MHA) or no change (no observable growth inhibition; TSA).

**Table 9:** *E. coli* streptomycin minimum inhibitory concentrations in agar with or without 120 ppm biochanin A

		Mueller Hinton				Tryptic Soy			
*E. coli* strain	Treatment	MIC^a^ Aerobic	CLSI Result^b^	MIC Anaerobic	CLSI Result	MIC Aerobic	CLSI Result	MIC Anaerobic	CLSI Result
ATCC 25922	Control	1.5 (1.5)	NE	32 (32)	NE	3 (2–3)	NE	64 (64)	NE
	120 ppm Biochanin A	1 (1)	NE	16 (16)	NE	8 (8)	NE	> 1024	NE
O157:H7 ATCC 51659	Control	16 (12–16)	NE	> 1024	NE	64 (64)	NE	> 1024	NE
	120 ppm Biochanin A	32 (32)	NE	384 (384)	NE	128 (128)	NE	> 1024	NE

^a^ Median MIC of antimicrobial range tested (0.064–1024 µg mL^− 1^)

^b^ CLSI breakpoint only established using disk diffusion for Enterobacterales

*NE* not established

## Discussion

Most beef cattle marketed in the United States are fed high grain diets during the finishing stage to obtain the desired carcass characteristics in a short time [1]. This feeding practice often results in acidification of the rumen leading to the development of parakeratosis and rumenitis, ultimately leading to pathogen infiltration into the blood [2]. The pathological risks to grain fed cattle often require the supplementation of antimicrobials into the feed, which substantially increases the risk of AMR adaptations in various pathogens, namely *E. coli* [3–5]. Given that antimicrobial administration in feedlot cattle has been associated with an increased incidence of AMR *E. coli*, it was pertinent that a resensitizing/synergistic agent be identified [9]. *E. coli* strain 51659 (O157:H7) has known resistance mechanisms to tetracycline (tetracycline class), sulfisoxazole (sulfonamide class), and streptomycin (aminoglycoside class), while 25922 is generally considered a drug-susceptible strain [19]. Therefore, determining the characteristics of BCA as either a resensitizing agent, synergistic compound, or potentially neither (for this organism and under the current testing conditions) was feasible using these two strains.

Some strains of *E. coli* possess efflux pumps active against the tetracyclines – e.g., tetA-G efflux pumps [20, 21] – the function of which could theoretically be blocked by a resensitizing agent, as has been demonstrated in other organisms resistant to antibiotics using efflux pumps [22]. For example, Hu et al. noted a decrease in gene expression of efflux pumps in *Xanthomonas axonopodis* (a soybean pathogen) when treated with < 100 ppm BCA [15]. Whereas Chovanova et al. demonstrated resensitization to tetracycline by *Staphylococcus epidermidis* strains and attributed this to the blocking of *tetK* efflux pump activity with sage essential oils [13]. These previous reports indicated that BCA may possess both resensitization and antimicrobial capabilities. However, the data reported here for tetracycline, minocycline, and doxycycline did not support these previous observations, as 120 ppm BCA treatment was found to either have no effect, a negative effect (increasing the MIC from intermediate to resistant), or a neutral effect (decreasing the MIC from resistant to intermediate based on CLSI breakpoint interpretation; Tables 1, 2 and 3). Note that the previous works and the data reported here were obtained using differing methodologies, i.e., microbroth dilutions (liquid medium; previous works) versus ETEST^®^ on agar (solid medium; current work). Indeed, a previous review of the microbiology literature demonstrated differing MIC determinations from liquid or solid media, with an emphasis on exposure to oxygen [23]. An additional review explored resistance mechanisms as a function of bacterial metabolism in general, including aerobic/anaerobic metabolism, and demonstrated that the suppression of several genes involved in basic metabolism altered susceptibility to antimicrobials [24].

Alternative to the tetracyclines, the common macrolide resistance mechanisms largely involve genes which code for enzymes that modify macrolide structures rendering them inactive, such as methylases (*erm* genes), esterases (*ere* genes), and phosphotransferases (*mph* genes) [25]. Importantly, some isolates of *E. coli* that possess these mutations had altered MICs to erythromycin, compared to controls, depending on which genes were present (16–1,024 µg mL^− 1^); this study used the disk diffusion method on agar but did not report exposure to oxygen or incubation temperature [25]. Additionally, the original report of azithromycin efficacy demonstrated that it was significantly more potent than erythromycin where it’s MIC for 90% of the *E. coli* isolates tested were ≤ 4 µg mL^− 1^ compared to erythromycin MICs of 16–128 µg mL^− 1^ [26]. This previous work determined the MICs of the Enterobacterales (e.g., *E. coli*) using the microbroth dilution method in Mueller Hinton broth under aerobic conditions incubated at 37 °C. Both sets of previously reported MICs are comparable with those reported here using MHA and TSA under aerobic conditions, also incubated at 37 °C: 1–2 µg mL^− 1^ MHA and 4–12 µg mL^− 1^ TSA for azithromycin, and 16 - >256 µg mL^− 1^ (no inhibition) MHA and 48–128 µg mL^− 1^ TSA for erythromycin (Tables 4 and 5, respectively). When grown anaerobically, however, neither macrolide possessed inhibitory qualities at the concentrations tested, indicating that oxygen exposure may have a substantial impact on MIC, which is consistent with previous work [23]. In fact, a previous study demonstrated that increased antibiotic tolerance was observed in anaerobic *Burkholderia* populations seemingly as a byproduct of an active anaerobic metabolism; an observation ameliorated upon oxygenation [27].

BCA addition increased the MIC of erythromycin when 25922 was grown aerobically in either MHA or TSA, and of 51659 in TSA but reduced the MIC in MHA, though this value was still within the range previously reported by other groups (Table 5) [26]. Therefore, similarly to the tetracyclines, 120 ppm BCA appeared to have either no impact, negative impact, or neutral impact on the MICs of the macrolides on *E. coli* strains 25922 and 51659.

BCA-mediated resensitization/synergy of/with fluoroquinolones has been performed using *Staphylococcus aureus* (Gram +) and *Ureaplasmas, *species (mollicutes). A previous report demonstrated synergistic relationships on *S. aureus* between BCA (128 ppm) and ciprofloxacin (128 µg mL^− 1^) using an agar disk diffusion method in MHA and microdilution method in Mueller-Hinton broth; both assays were incubated aerobically at 37 °C [16]. A second report noted synergy between BCA and the fluoroquinolones ciprofloxacin and ofloxacin where BCA addition enhanced the inhibitory activity of the antibiotics on *Ureaplasma* species significantly [28]. Our data demonstrates that *E. coli* was susceptible to ciprofloxacin regardless of 120 ppm BCA addition. Contrary to the previous reports, BCA when it elicited a change in MIC at all, generally increased the MIC modestly without changing the CLSI interpretation, i.e., susceptible (Table 6). It seems that *E. coli* (Gram -) fluoroquinolone resistance (MICs of 2–500 µg mL^− 1^ for ciprofloxacin) is primarily governed by mutations in genes coding for gyrase (*gyrA* and *gyrB*) and topoisomerase IV (*parC* and *parE*), with some evidence for increased prevalence of a multi-drug efflux pump (*acrA*) [29]. This study reported that their wild-type isolates had tested MICs between 0.004 and 0.094 µg mL^− 1^ using ETEST^®^ on LB agar supplemented with 0.2% arabinose, which were comparable to our results in both MHA and TSA with a few exceptions when *E. coli* was grown aerobically in TSA where the MIC reached 0.19 µg mL^−1^ (Table 6). Given the previous reports on the MIC impact of resistance mechanisms in *E. coli*, it appears that neither strain used in this study possesses (whether adapted or otherwise) these necessary mutations. The presence of 120 ppm BCA in the growth medium, therefore, did not act as a resensitizing agent nor appear to perform as a synergistic compound when ciprofloxacin was administered.

The antifolate and sulfonamide mechanism of action is dependent on transport into the cell as it interferes with the activity of the enzymes dihydrofolate reductase (DHFR; trimethoprim) and dihydropteroate synthase (DHPS; trimethoprim/sulfamethoxazole) [30, 31]. Both trimethoprim and trimethoprim/sulfamethoxazole are approved first-line antibiotics for Gram (+) and (-) bacterial infections, including urinary tract infections caused by *E. coli* [31, 32]. While efflux pumps are common resistance mechanisms, those employed in the organisms resistant to trimethoprim and sulfamethoxazole seem to focus on modifications to the drug target itself, i.e., mutations in the *dfr* (encoding DHFR), *fo*l and *sul* (encoding DHPS) genes [31]. Therefore, resensitizing agents dependent on the action of efflux pumps might not be useful given the resistance mechanism. Indeed, the data reported here demonstrate that when BCA had an impact on *E. coli* sensitivity to the antibiotics, it was negative, i.e., increased the MIC to > 32 µg mL^− 1^ (Tables 7 and 8). However, the data indicate culture condition differences in sensitivity as well; namely, media composition and the absence of oxygen. These observations are in alignment with previous consensus that nutritional availability (media type) and the presence or absence of oxygen can impact MIC of an antimicrobial substance [23]. The data presented here indicate that the presence of 120 ppm BCA in agar did not elicit synergistic antimicrobial action with the antifolate/sulfonamide against either strain of *E. coli*.

The aminoglycoside, streptomycin, is not a first-line treatment for *E. coli* infections, and therefore the CLSI breakpoints have not been established using ETEST^®^ strips. However, streptomycin can be used to treat some *E. coli* infections, and the species is known to have resistance mechanisms to this antibiotic despite not being a commonly used treatment. Therefore, the MICs of streptomycin on *E. coli* 25922 and 51659 under various culture conditions were reported here (Table 9). Previous data indicate that the resistance genes (*strA*, *strB*, and *aadA*), sometimes present in the genomes of *E. coli*, greatly impact the MIC of streptomycin in these isolates [33]. For example, most isolates with neither gene group or the *aadA* gene only had MICs of 16 µg mL^− 1^, whereas isolates with all three resistance genes had MICs ≥ 128 µg mL^− 1^ [33]. While the culture conditions of the previous experiment were unknown, the MICs are comparable to those reported in the present study, which supports the presence of resistance genes, especially in strain 51659, or *E. coli* O157:H7 (Table 9). Isolates of *E. coli* O157:H7 are known to possess resistance genes, and ATCC 51659 is characterized as resistant to streptomycin [19, 34, 35]. However, 51659 resistance to streptomycin couldn’t be measured clinically using the ETEST^®^ strips as there is currently no established CLSI breakpoint (CLSI M100). Regardless, growth of 51659 was uninhibited by streptomycin when cultured anaerobically, except in MHA when 120 ppm BCA had been added where it’s MIC decreased to 384 µg mL^− 1^. Additionally, there were measurable MICs of 51659 in both MHA and TSA when cultured aerobically, so resistance as characterized by a lack of growth inhibition could not be determined. However, these MICs may still be defined as resistant if a breakpoint were to be established for this antibiotic against the Enterobacterales.

The effect of BCA addition on MIC remained similar for all the antibiotic classes tested here on *E. coli* strains 25922 and 51659: either it had no effect, a negative effect, or a neutral effect (decreasing the MIC, but to a non-clinically relevant degree). In the case of the tetracycline antibiotics, neither media type nor exposure to oxygen had a clinically meaningful impact on MIC (Tables 1, 2 and 3). The known resistant strain 51659, was largely unaffected by BCA addition which generally had no effect on its resistance to the tetracycline antibiotics whether through action as a resensitizing or synergistic agent. For the macrolides, the presence of oxygen seemed to be the most significant factor influencing MIC of both strains of *E. coli* tested here and the presence of BCA did not appear to have a significant impact on those MICs (Tables 4 and 5). Ciprofloxacin MICs were clinically unaffected by media type (composition), atmospheric conditions (oxygen exposure), nor 120 ppm BCA addition and *E. coli* remained susceptible to the antibiotic under all testing conditions (Table 6). The MICs of trimethoprim/sulfamethoxazole against *E. coli* 25922 were clinically affected by the presence of BCA in the agar, in that its presence appeared to make the organism resistant to the antibiotic combination regardless of media type but only in the absence of oxygen (Table 7). Whereas 25922 appeared to be clinically impacted by the composition of its growth medium where it was resistant under both atmospheric conditions when cultured on TSA and exposed to only trimethoprim (Table 8). *E. coli* 51659 trimethoprim/sulfamethoxazole and trimethoprim MICs generally appeared clinically impacted by media type (TSA) coupled with anaerobic growth conditions where it was resistant (Tables 7 and 8). However, 51659 trimethoprim MIC was clinically impacted by the presence of BCA under anaerobic conditions when grown on MHA where it became resistant after being susceptible without BCA. For each strain and each media type, *E. coli* growth inhibition by streptomycin seemed to be largely dependent on oxygen exposure, where either the MICs increased drastically or there was no growth inhibition at the concentrations tested when it was cultured anaerobically (Table 9). BCA addition, similarly to the other antibiotics tested, had limited impact that could not be defined as resensitization or synergy in a clinically relevant manner when *E. coli* was exposed to streptomycin.

## Conclusions

While there appears to be evidence for resensitizing/synergistic qualities of BCA for all manner of antibiotic classes in Gram (+) and mollicutes organisms, the Gram (-) *E. coli* 51659 and 25922 were less susceptible to the isoflavone’s hypothetical benefits. This could be a function of cell morphology, i.e., presence of a cell wall, or it could be largely dependent on methodology, such as microbroth dilution or agar (disk diffusion versus ETEST^®^), or even still the presence or absence of oxygen in conjuncture with these other potentially critical differences. Further study may be necessary using different methodologies to determine if BCA truly lacks value as a possible resensitization/synergistic compound for resistant strains of *E. coli*. However, these data indicate that BCA likely does not possess a synergistic effect with the antibiotics in this study and therefore lacks use for control of *E. coli* despite previous works demonstrating its usefulness for other resistant Gram (+) or mollicutes species. Regardless, the data also demonstrates the impact culture conditions have on the MICs of various classes of antibiotics, some of which are important first-line treatments for *E. coli* infections, such as trimethoprim and trimethoprim/sulfamethoxazole. These differences in susceptibilities could be important to consider when treating anaerobic *E. coli* infections where the nutrients available may allow the organism to more robustly resist the inhibitory action of the therapeutics administered.

## Data Availability

All data generated or analyzed during this study are included in this published article.
